# Device-Related Complications in Transvenous Versus Subcutaneous Defibrillator Therapy During Long-Term Follow-Up: The PRAETORIAN-XL Trial

**DOI:** 10.1161/CIRCULATIONAHA.125.074576

**Published:** 2025-04-25

**Authors:** Louise R.A. Olde Nordkamp, Jolien A. de Veld, Abdul Ghani, Jürgen Kuschyk, Hendrik Bonnemeier, Kerstin Bode, Lucas V.A. Boersma, Anouk de Weger, Jonas S.S.G. de Jong, Ward P.J. Jansen, Marco Alings, Nick Bijsterveld, Mikhael F. El-Chami, Rypko J. Beukema, Kevin Vernooy, Berit T. Philbert, Petr Neuzil, Peter Nordbeck, Jurren M. van Opstal, Cornelis P. Allaart, David J. Wright, Michael Knaut, Timothy R. Betts, Zachary I. Whinnett, Pier D. Lambiase, Joris R. de Groot, Alexandru B. Chicos, Dimitry Nemirovksy, Stefan Kääb, Suneet Mittal, Alida E. Borger van der Burg, Leonard A. Dijkshoorn, Shari Pepplinkhuizen, Willeke van der Stuijt, Jose M. Dizon, Marc A. Miller, Elijah R. Behr, Martin C. Burke, Kirsten M. Kooiman, Anne-Floor B.E. Quast, Tom F. Brouwer, Arthur A.M. Wilde, Lonneke Smeding, Reinoud E. Knops, on behalf of the PRAETORIAN-XL Investigators

**Affiliations:** 1 Department of Cardiology, University of Amsterdam, Amsterdam, the Netherlands (L.R.A.O.N., J.A.d.V., L.V.A.B., A.d.W., J.R.d.G., L.A.D., S.P., W.v.d.S., K.M.K., A.B.E.Q., T.F.B., A.A.M.W., L.S., R.E.K.).; 2 Department of Cardiology, Isala Heart Centre, Zwolle, the Netherlands (A.G.).; 3 First Department of Medicine, University Medical Center Mannheim, Mannheim, Germany (J.K.).; 4 German Center for Cardiovascular Research Partner Site Heidelberg, Mannheim, Germany (J.K.).; 5 Christian Albrechts University Kiel, Kiel, Germany (H.B.).; 6 Department of Electrophysiology, Heart Center at University of Leipzig, Leipzig, Germany (K.B.).; 7 Department of Cardiology, St. Antonius Hospital, Nieuwegein, the Netherlands (L.V.A.B.).; 8 Department of Cardiology, OLVG, Amsterdam, the Netherlands (J.S.S.G.s.J.).; 9 Department of Cardiology, Tergooi MC, Blaricum, the Netherlands (W.P.J.J.).; 10 Department of Cardiology, Amphia Hospital, Breda, the Netherlands (M.A.).; 11 Werkgroep Cardiologische Centra Nederland, Utrecht, the Netherlands (M.A.).; 12 Department of Cardiology, Flevoziekenhuis, Almere, the Netherlands (N.B.).; 13 Division of Cardiology, Section of Electrophysiology, Emory University, Atlanta, GA (M.F.E.-C.).; 14 Department of Cardiology, Radboud University Medical Center, Nijmegen, the Netherlands (R.J.B.).; 15 Department of Cardiology, Cardiovascular Research Institute Maastricht, Maastricht University Medical Center, Maastricht, the Netherlands (K.V.).; 16 Department of Cardiology, The Heart Centre, Rigshospitalet, University of Copenhagen, Copenhagen, Denmark (B.T.P.).; 17 Department of Cardiology, Homolka Hospital, Prague, Czech Republic (P. Neuzil).; 18 University and University Hospital Würzburg, Würzburg, Germany (P. Nordbeck).; 19 Medical Spectrum Twente, Enschede, the Netherlands (J.M.v.O.).; 20 Department of Cardiology and Amsterdam Cardiovascular Sciences (ACS), Amsterdam UMC, Location VUMC, Amsterdam, the Netherlands (C.P.A.).; 21 Liverpool Heart and Chest Hospital, Liverpool, United Kingdom (D.J.W.).; 22 Heart Surgery, Heart Center Dresden, Carl Gustav Carus Medical Faculty, Dresden University of Technology, Dresden, Germany (M.K.).; 23 Oxford Biomedical Research Centre, Oxford University Hospitals NHS Trust, Oxford, United Kingdom (T.R.B.).; 24 National Heart and Lung Institute, Imperial College London, London, United Kingdom (Z.I.W.).; 25 Office of the Director of Clinical Electrophysiology Research and Lead for Inherited Arrhythmia Specialist Services, University College London and Barts Heart Centre, London, United Kingdom (P.D.L.).; 26 Division of Cardiology, Northwestern Memorial Hospital, Northwestern University, Chicago, IL (A.B.C.).; 27 Department of Medicine, Englewood Hospital and Medical Center, Englewood, NJ (D.N.).; 28 Department of Medicine I, Ludwig-Maximillians University Hospital, München, Germany (S.K.).; 29 German Center for Cardiovascular Research, Munich Heart Alliance, Munich, Germany (S.K.).; 30 The Valley Health System, Ridgewood, NJ (S.M.).; 31 Medisch Centrum Leeuwarden, Leeuwarden, the Netherlands (A.E.B.v.d.B.).; 32 Department of Medicine – Cardiology, Columbia University Irving Medical Center, New York, NY (J.M.D.).; 33 Icahn School of Medicine at Mount Sinai, Mount Sinai Hospital, New York, NY (M.A.M.).; 34 St George’s University of London, London, United Kingdom (E.R.B.).; 35 St George’s University Hospitals NHS Foundation Trust, London, United Kingdom (E.R.B.).; 36 CorVita Science Foundation, Chicago, IL (M.C.B.).; 37 European Reference Network for Rare, Low Prevalence and Complex Diseases of the Heart: ERN GUARD-Heart (A.A.M.W.); Department of Electrophysiology, Catharina Hospital, Eindhoven, the Netherlands; Amsterdam UMC, University of Amsterdam, Heart Center, Department of Cardiology, Amsterdam Cardiovascular Sciences Heart Failure & Arrhythmias, Amsterdam, The Netherlands; Department of Internal Medicine and Cardiology, University Medical Center Schleswig-Holstein, Kiel, Germany; Department of Cardiology, OLVG, Amsterdam, the Netherlands; Department of Cardiology, Queen Elisabeth Hospital Liverpool, Liverpool, United Kingdom; Department of Cardiology, Medisch Centrum Alkmaar, Alkmaar, The Netherlands; Amsterdam UMC, University of Amsterdam, Heart Center, Department of Cardiology, Amsterdam Cardiovascular Sciences Heart Failure & Arrhythmias, Amsterdam, The Netherlands.

**Keywords:** complications, defibrillators, implantable, electrophysiology

## Abstract

**BACKGROUND:**

The PRAETORIAN trial (A Prospective, Randomized Comparison of Subcutaneous and Transvenous Implantable Cardioverter Defibrillator Therapy) investigated the efficacy and safety of the subcutaneous implantable cardioverter defibrillator (S-ICD) compared with a transvenous ICD (TV-ICD) and showed noninferiority of the S-ICD with regard to the composite end point of device-related complications and inappropriate shocks after 49.1 months. Complications associated with transvenous leads are expected to occur after longer follow-up. The PRAETORIAN-XL trial aims to investigate whether the S-ICD is superior to the TV-ICD with respect to device-related complications at 8-year follow-up.

**METHODS:**

The PRAETORIAN trial randomized patients with a class I or IIa indication for ICD therapy without the need for pacing to either S-ICD or TV-ICD among 39 centers in the United States and Europe between March 2011 and January 2017. The follow-up was extended after 49.1 months by an additional 4 years for the PRAETORIAN-XL trial. The primary end point was the composite of all device-related complications. Complications could be related or unrelated to the lead and minor or major, with major complications being those requiring an invasive intervention. End points were analyzed according to the modified intention-to-treat principle using a Fine-Gray subdistribution hazards model to account for competing risks. An as-treated analysis was performed using a Cox proportional hazards model with device type as time-dependent variable.

**RESULTS:**

Patients were randomized to S-ICD (n=426) and TV-ICD (n=423). Twenty-one percent of the S-ICD group versus 18% of the TV-ICD group were women. The median age at implantation was 63 (interquartile range, 54–69) years for the S-ICD and 64 (interquartile range, 56–69) years for the TV-ICD. After a median follow-up of 87.5 months, all device-related complications (major and minor combined) were not significantly different in the modified intention-to-treat analysis (subdistribution hazard ratio, 0.73 [95% CI, 0.48–1.12]); *P*=0.15). However, TV-ICD patients more often had a major complication or lead-related complication (*P*=0.03 and *P*<0.001, respectively). Moreover, the as-treated analysis showed significantly more complications in patients with a TV-ICD compared with an S-ICD (hazard ratio, 0.64 [95% CI, 0.41–0.99]; *P*=0.047).

**CONCLUSIONS:**

The PRAETORIAN-XL trial demonstrated that there was no significant difference between the S-ICD and TV-ICD in all device-related complications during long-term follow-up. However, the TV-ICD carries a higher risk of major and lead-related complications compared with S-ICD therapy. The S-ICD should therefore be considered for all patients without a pacing indication who are evaluated for ICD therapy.

**REGISTRATION:**

URL: https://www.clinicaltrials.gov; Unique identifier: NCT01296022.

**Clinical Perspective**

**What Is New?**

- This is the first randomized comparison of subcutaneous and transvenous implantable cardioverter defibrillator (ICD) complications during long-term follow-up.
- Patients who receive a transvenous ICD have an increased risk of lead-related and major complications.
- The high generator change rate in the subcutaneous ICD does not lead to a higher risk of device-related complications compared with the transvenous ICD.

**What Are the Clinical Implications?**

- The subcutaneous ICD should be considered for all ICD patients without an indication for pacing.

Implantable cardioverter defibrillators (ICDs) are a safe and effective therapy for the prevention of sudden cardiac death.^1–3^ For decades, conventional transvenous ICDs (TV-ICDs) were the standard of care, but these devices are associated with a risk of complications related to transvenous leads, such as infection, pneumothorax, and lead dysfunction.^4,5^ Subcutaneous ICDs (S-ICDs) have a totally extravascular design and were developed to overcome the risk of these lead-related complications.^6^

To compare the TV-ICD with the S-ICD, the multicenter randomized PRAETORIAN trial (A Prospective, Randomized Comparison of Subcutaneous and Transvenous Implantable Cardioverter Defibrillator Therapy) was conducted.^7^ In this trial, 849 patients with a class I or IIa indication for ICD therapy without the need for pacing were randomized to either S-ICD or TV-ICD therapy, and patients were followed for a median duration of 49.1 months. The trial showed that the S-ICD was noninferior to TV-ICDs with regard to the composite primary end point of device-related complications and inappropriate shocks. Subsequently, a secondary analysis of the PRAETORIAN trial showed that complications in the TV-ICD arm were more severe, as they required invasive interventions more frequently.^8^

Early concerns of increased numbers of inappropriate shocks resulting from oversensing since the introduction of the S-ICD have been mitigated by optimization of programming and new software algorithms.^9,10^ Complications with transvenous leads, especially lead failure and lead infections, generally continue to rise during long-term follow-up.^11,12^ To compare the S-ICD with the TV-ICD with regard to device-related complications over time, the follow-up of participants in the PRAETORIAN trial was extended for an additional 48 months: the PRAETORIAN-XL trial.

## METHODS

The data that support the findings of this study are available from the corresponding author upon reasonable request.

### Patient Population and Trial Overview

Between March 2011 and January 2017, 849 patients (426 S-ICD and 423 TV-ICD) with a class I or IIa indication for ICD therapy were included in 39 centers across Europe and the United States. Complete rationale and study design were published previously.^7,13^ Key exclusion criteria involved failure of S-ICD screening, indications for bradycardia pacing, or expected benefit of antitachycardia pacing (ATP). Patients were randomized at a 1:1 ratio to either undergo S-ICD or TV-ICD implantation. All transvenous devices were single-chamber ICDs unless a dual-chamber device was deemed necessary for arrhythmia discrimination. Programming was mandated per protocol, and strategies were comparable between treatment groups.^13^ For the initial trial, patients were followed for a median of 49.1 months, with the end of follow-up on December 1, 2019. For the extended PRAETORIAN-XL trial, all patients active at the end of the trial were asked to provide consent for an additional 48 months of follow-up until December 1, 2023. Patients who did not consent to the extended PRAETORIAN-XL trial were censored from December 1, 2019. Patients who gave consent were observed without any additional study-specific interventions. The PRAETORIAN-XL protocol amendment was approved by the local institutional medical ethics committees.

### Primary End Point of PRAETORIAN-XL

The primary end point of the PRAETORIAN-XL trial was the composite of device-related complications, as defined in the initial PRAETORIAN protocol. These complications included the following: device infection that led to extraction of the lead or generator; pocket hematoma resulting in drainage, blood transfusion, or prolongation of hospitalization; device-related thrombotic events; pneumo- or hemothorax resulting in intervention or prolonged hospitalization; cardiac perforation or tamponade; lead repositioning or replacement; and other complications related to the ICD that led to medical or surgical intervention. Complications were considered major if they resulted in an invasive intervention and minor if they did not. Additional procedures as a result of the development of a pacing indication or progression of heart failure were not included as a device-related complication, as these were deemed to be attributable to disease progression rather than the device itself. Additionally, early and expected battery depletion were not included as primary end points. All complications were adjudicated by device specialists familiar with both the S-ICD and TV-ICD. They were blinded for randomization group but, by nature of the treatment, not for device type. As secondary end points, mortality and the incidence of major adverse cardiac events were captured, the latter being defined as cardiac death, myocardial infarction, percutaneous coronary intervention, coronary artery bypass grafting, or any valve surgery. In addition, the incidence of cardiac decompensation was collected.

### Statistical Analysis

Descriptive statistics are presented as mean with standard deviation (SD) or as median with interquartile range (IQR) for continuous variables or as numbers and proportions for categorical variables. The primary analysis for all primary and secondary end points was performed according to the modified intention-to-treat principle, in which patients were analyzed by the randomization group to which they were allocated, and all complications were included, also if they occurred in another device type than the one to which the patient was randomized. Patients were excluded from this analysis if they did not receive either device after randomization or if they underwent randomization in error. For this modified intention-to-treat analysis, a Fine-Gray subdistribution hazard model was used to account for the competing risks of death and loss to follow-up. Randomization group was used as a covariate. Effect sizes are expressed using subdistribution hazard ratios (sHR) with corresponding 95% CI. To illustrate the event rates over time, 8-year estimated cumulative incidences derived from the Fine-Gray model were generated and compared using Gray’s test. This test evaluates the cumulative incidence function in the presence of competing risks. For the as-treated analysis for the primary end point of all device-related complications, a time-dependent Cox proportional hazards model was used, with ICD type as the time-dependent variable. In the as-treated analysis, patients were censored after a cardiac resynchronization therapy defibrillator (CRT-D) upgrade, and complications in a CRT-D were excluded. The effect size in this analysis is expressed using a hazard ratio (HR) with corresponding 95% CI. To generate 8-year estimated cumulated incidence curves and a *P* value in this analysis, a Wald’s test was used. Additional information about the statistical analyses is provided in the Supplemental Appendix. Analyses were performed using R Software version 4.4.3 (RStudio PBC) and SPSS version 28.

## RESULTS

### Patient Characteristics

**Figure 1.**
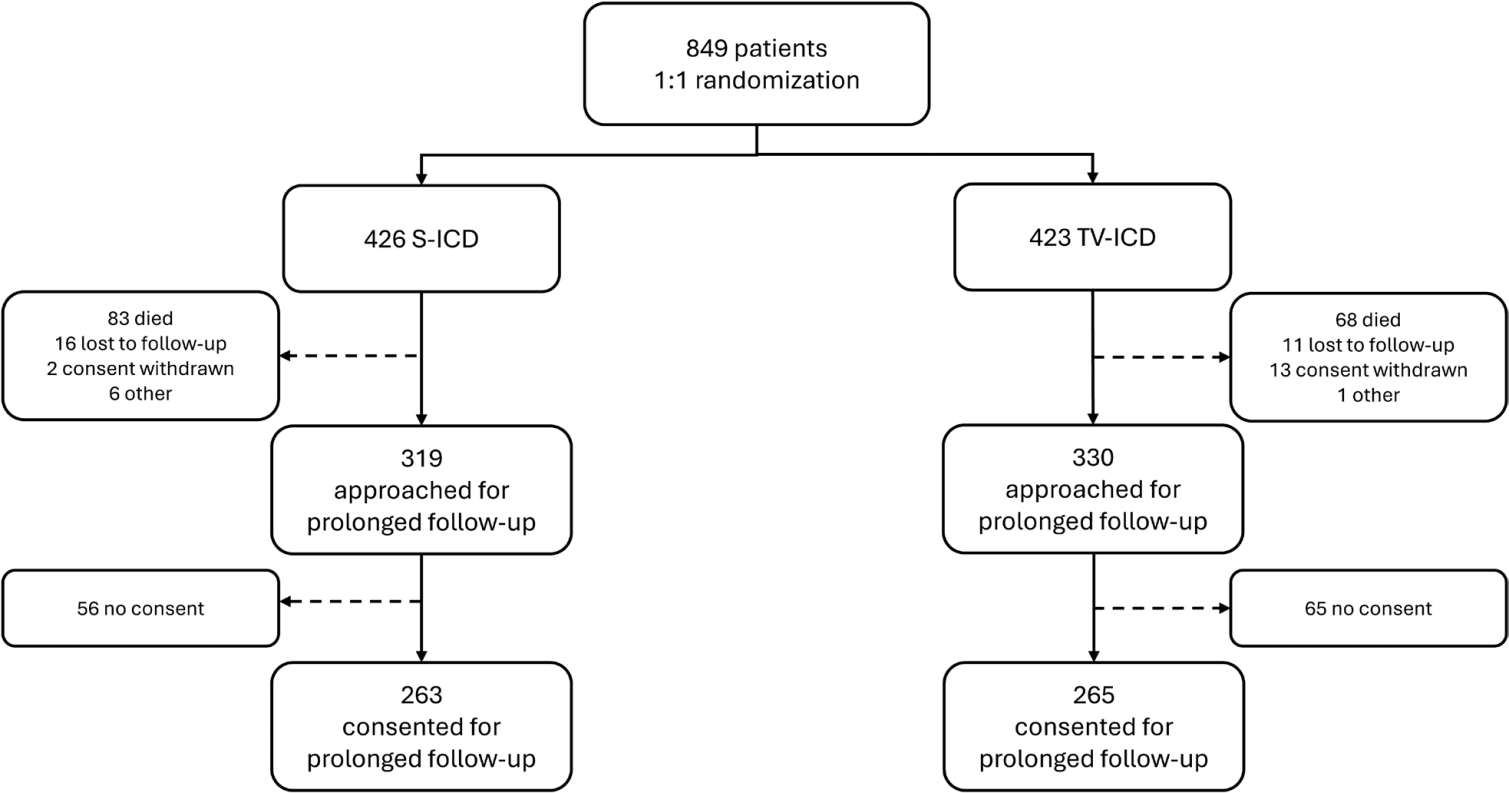
**Flowchart for composition of the study cohort.** S-ICD indicates subcutaneous implantable cardioverter defibrillator; and TV-ICD, transvenous implantable cardioverter defibrillator.

At the start of the PRAETORIAN trial, 849 patients were included, of whom 426 were randomized to S-ICD and 423 to TV-ICD. Table 1 provides the patient characteristics at baseline. In short, the median age at implantation was 63 (IQR, 54–69) years in the S-ICD group and 64 (IQR, 56–70) in the TV-ICD group, 21% were women in the S-ICD group and 18% in the TV-ICD group (sex assigned at birth), and the most common diagnosis was an ischemic cardiomyopathy in both arms (68% for S-ICD and 70% for TV-ICD). A total of 649 patients were approached for participation in the extension of the PRAETORIAN trial, the PRAETORIAN-XL trial, of whom 263 of 319 (82%) of the S-ICD patients and 265 of 330 (80%) of the TV-ICD patients provided written informed consent for this extended follow-up. A flowchart of the study cohort composition is shown in Figure 1. Information about the baseline characteristics of patients who were approached for PRAETORIAN-XL is presented in Table S1. Patient loss did not lead to imbalances in clinical characteristics between study arms (Table S2).

**Table 1.**
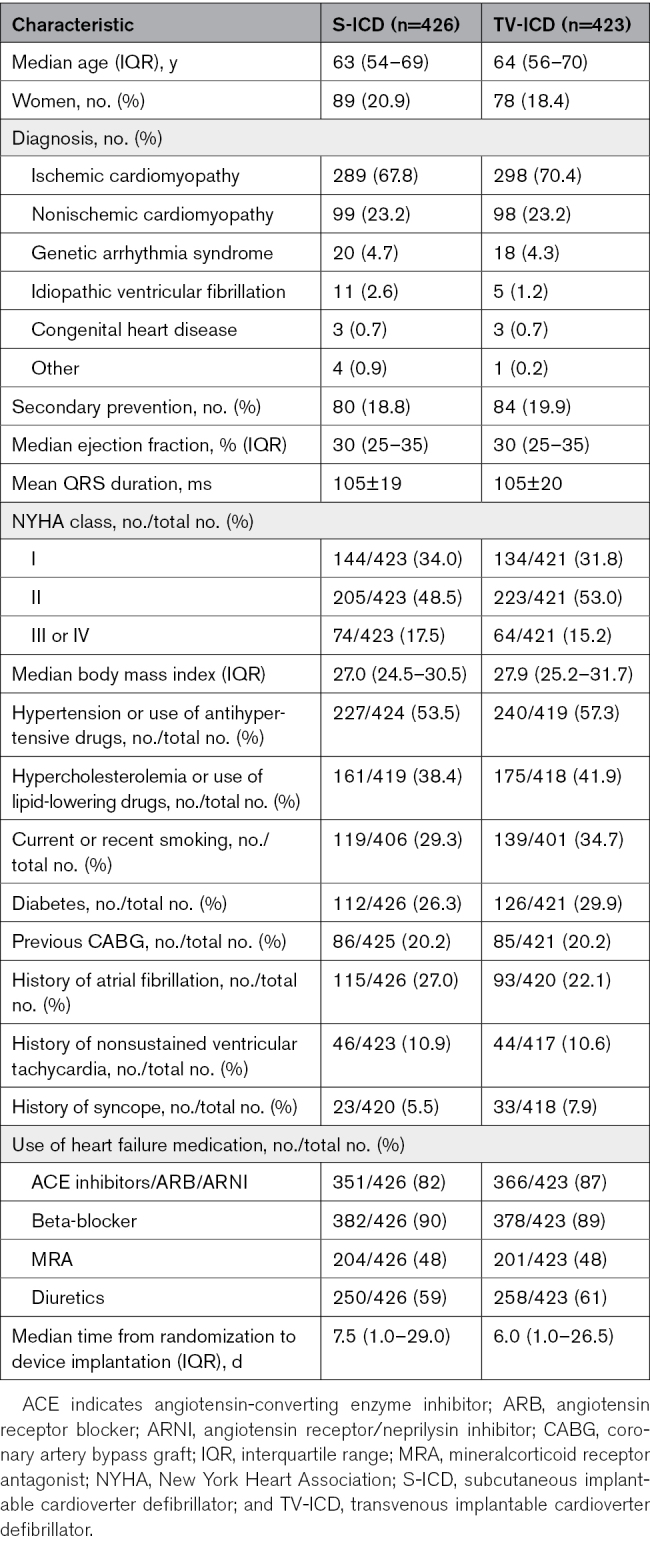
Patient Characteristics in the PRAETORIAN-XL Trial

### Minor and Major Complications

**Figure 2.**
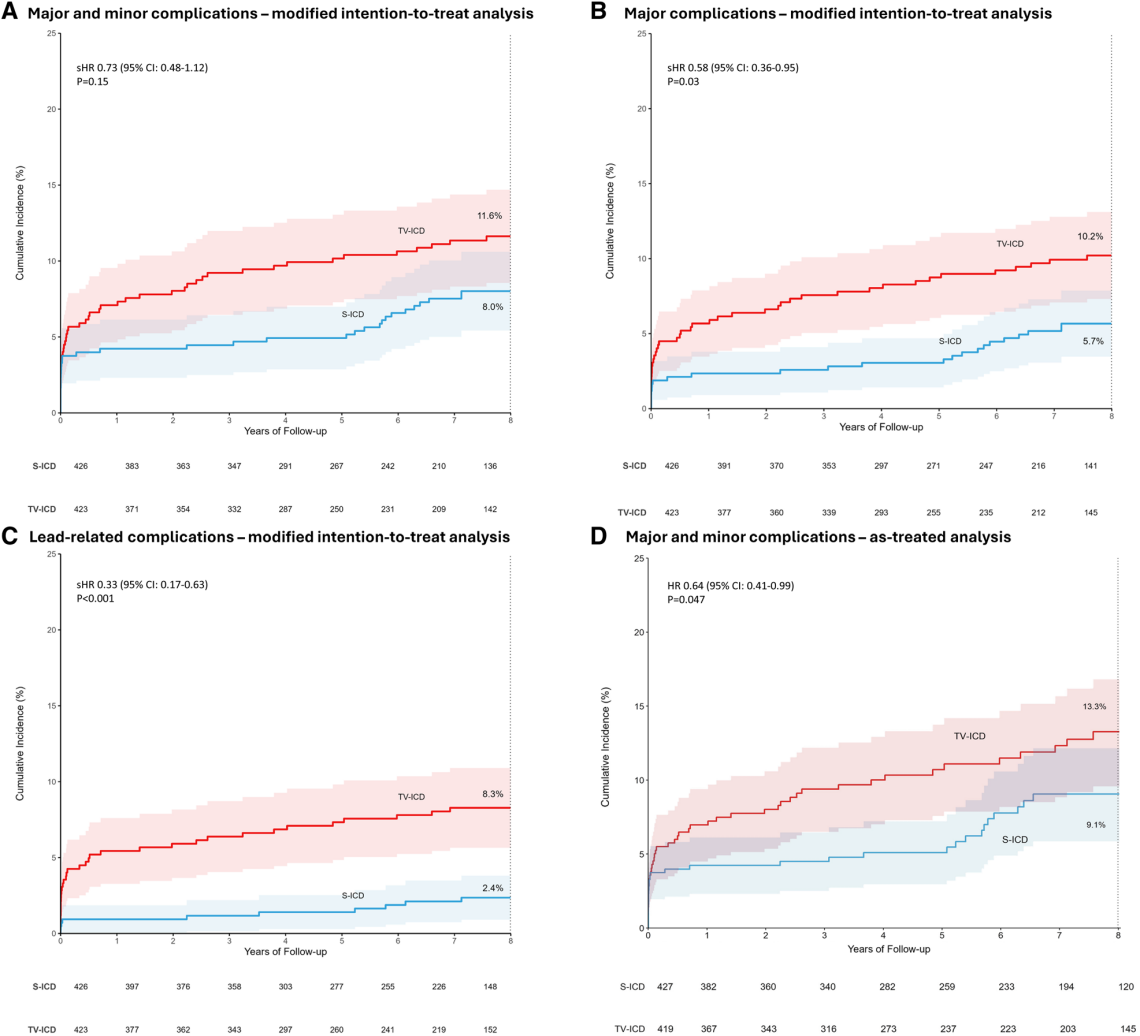
**Eight-year estimated cumulative incidences of device-related complications** Shown are the results of the following analyses. **A**, Modified intention-to-treat of all complications. In the subcutaneous implantable cardioverter defibrillator group, an increase in cumulative incidence was reported after 5 years of follow-up. In this group, 16 of 37 patients with a complication experienced their first complication after 5 years. In these patients, 10 of 16 (63%) complications emerged as a result of: (1) complication of generator replacement, (2) defibrillation test failure during generator replacement, (3) re-evaluation and subsequent repositioning of device position during generator replacement, and (4) re-evaluation of the device type at the time of generator replacement, followed by a subsequent complication. **B**, modified intention-to-treat of major complications. **C**, Modified intention-to-treat of lead-related complications. **D**, As-treated of all complications. HR indicates hazard ratio; sHR, subdistribution hazard ratio; S-ICD, subcutaneous implantable cardioverter defibrillator; and TV-ICD, transvenous implantable cardioverter defibrillator.

The median follow-up duration was 87.5 (IQR, 44.9–105.2) months in the S-ICD group and 87.4 (IQR, 46.1–103.8) months in the TV-ICD group (*P*=0.80). Of the 98 device-related complications in this study, 43 occurred in 37 patients in the S-ICD group, and 55 occurred in 49 patients in the TV-ICD group (8-year estimated cumulative incidence 8.0% and 11.6%, respectively; sHR, 0.73 [95% CI, 0.48–1.12]; *P*=0.15; Figure 2A; Table 2 ;Figure S1). Of the 98 complications, 23 occurred during the extended follow-up.

**Table 2.**
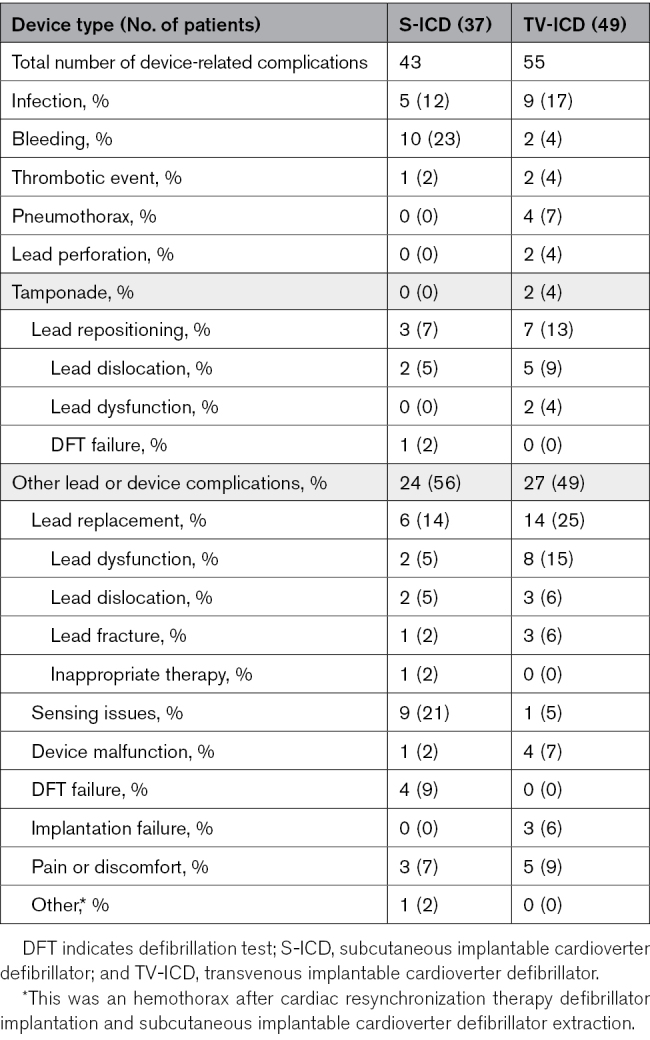
Number of Device-Related Complications

In total, 30 of 43 complications in the S-ICD group and 48 of 55 complications in the TV-ICD group were major (Table S3). Patients in the TV-ICD group had a significantly higher rate of major complications compared with patients in the S-ICD group (8-year estimated cumulative incidence 5.7% for the S-ICD group and 10.2% for the TV-ICD group; sHR, 0.58 [95% CI, 0.36–0.95]; *P*=0.03; Figure 2B).

The most common complication, including both major and minor events, was bleeding (10 of 43) in the S-ICD group and lead replacement (14 of 55) in the TV-ICD group (Table 2). Among major complications, the most frequent was a sensing issue (7 of 30) in the S-ICD group and lead replacement (14 of 47) in the TV-ICD group. Actions as a result of major complications are shown in Figure S2.

### Lead-Related Complications

There were significantly more patients in the TV-ICD group (35 of 49) with a lead-related complication compared with the S-ICD group (12 of 37; 8-year estimated cumulative incidence 2.4% for the S-ICD group and 8.3% for the TV-ICD group; sHR, 0.33 [95% CI, 0.17–0.63]; *P*<0.001; Figure 2C).

### Complications per Device Type

**Figure 3.**
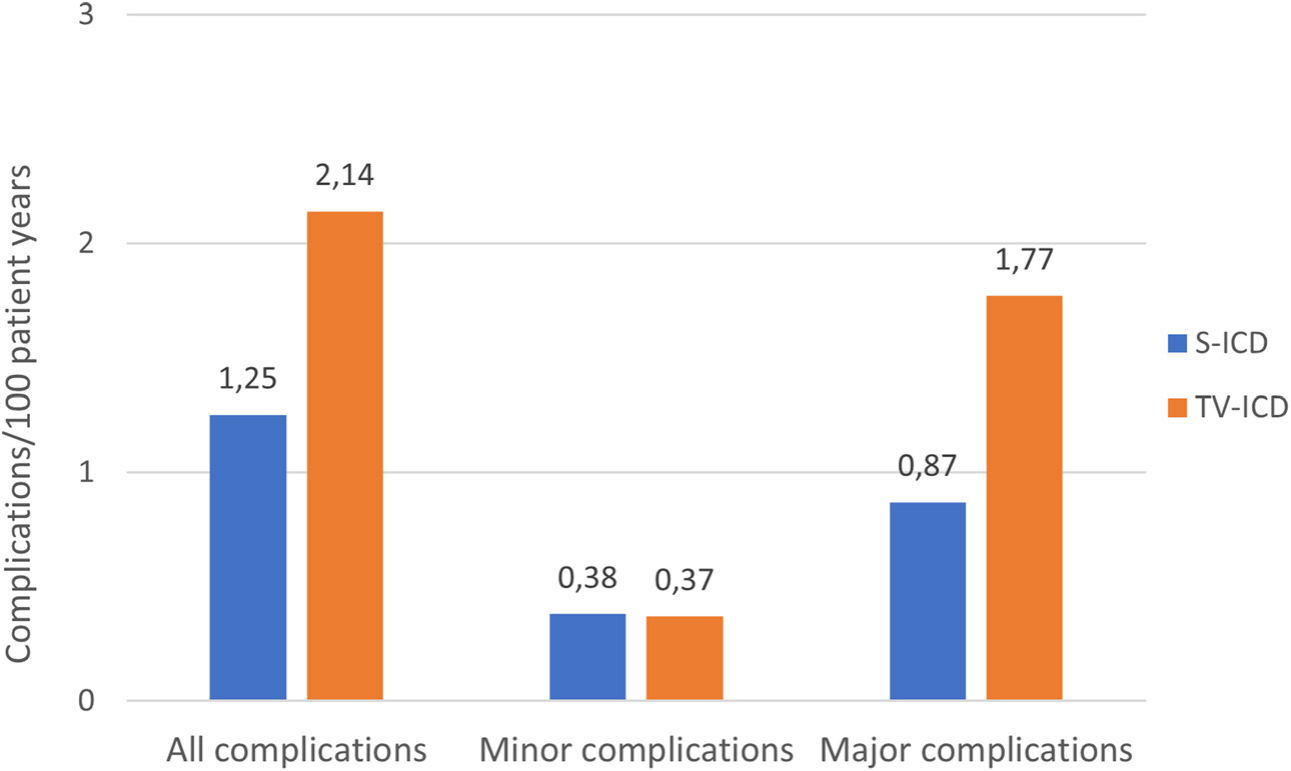
**Complications stratified by device in which it occurred.** The number of complications in the transvenous implantable cardioverter defibrillator was nearly twice as high compared with the S-ICD. This was mainly because of major complications, leading to an invasive intervention. S-ICD indicates subcutaneous implantable cardioverter defibrillator; and TV-ICD, transvenous implantable cardioverter defibrillator

Multiple complications occurred in patients who, during the course of the study, received a device different from the one to which they were randomized. In the S-ICD group, 5 of 43 (12%) complications occurred in patients implanted with a TV-ICD and 5 of 43 (12%) complications occurred in patients who were upgraded to a CRT-D, while 2 of 55 (4%) complications in the TV-ICD group occurred in patients who were upgraded to a CRT-D and none in patients who were implanted with an S-ICD. Of the 98 complications in this trial, 33 (34%) occurred with an S-ICD, 58 (59%) with a TV-ICD, and 7 (7%) with a CRT-D (Table S4). The number of complications per 100 patient years in S-ICD and TV-ICD are presented in Figure 3. As a result, the as-treated analysis showed a significantly higher incidence of device-related complications in patients implanted with a TV-ICD compared with patients implanted with an S-ICD (8-year estimated cumulative incidence 9.1% for the S-ICD and 13.3% for the TV-ICD; HR, 0.64 [95% CI, 0.41–0.99]; *P*=0.047; Figure 2D).

### Changes in Device Type

In total, 52 patients in the S-ICD arm and 43 patients in the TV-ICD arm were implanted with another device type during the course of the study (Table 3). In the S-ICD arm, 27 patients were upgraded to a CRT-D and 25 switched to a TV-ICD. In the TV-ICD group, 32 patients were upgraded to a CRT-D and 11 switched to an S-ICD. All CRT-D upgrades in the TV-ICD group and 22 of 27 CRT-D upgrades in the S-ICD group were attributable to progression of heart failure. The median time from implantation of the initial device to CRT-D resulting from heart failure was 45 months (IQR, 24–78). In the S-ICD group, 11 patients switched to a TV-ICD, and one converted to a CRT-D, because of an indication for bradycardia pacing, after a median time of 40 months (IQR, 22–79) months after implantation. In 3 patients in the S-ICD group, an indication for ATP was the reason for the switch in device, which resulted in one CRT-D and 2 TV-ICDs. In the TV-ICD group, apart from CRT indications, the most common reason for a device switch was preference of the patient, resulting in S-ICD implantation.

**Table 3.**
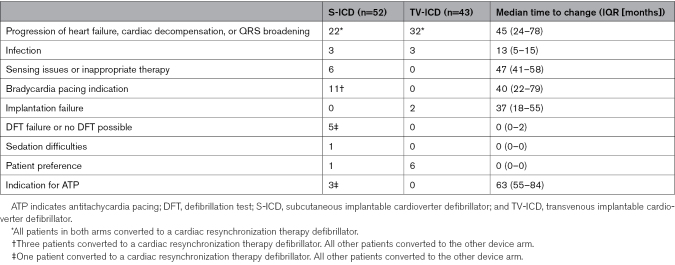
Reasons for Changes in Device Type

### Generator Replacements As a Result of Battery Depletion

In the S-ICD group, 199 generator replacements in 197 (46%) patients occurred because of battery depletion, whereas this occurred 39 times in 39 (9%) patients in the TV-ICD group. Of these generator replacements, 48 of 199 and 7 of 39 were for premature battery depletion in the S-ICD group and TV-ICD group, respectively (Table S5). The median service life from implantation to first replacement was 72 (IQR, 66–78) months in the S-ICD group and 99 (IQR, 74–117) months in the TV-ICD group. All early battery depletions were attributable to an advisory field safety notice. Of the device-related complications reported in this study, 4 occurred directly after a generator replacement in an S-ICD patient. In 3 cases, DFT failure occurred after the generator replacement, which led to subsequent intervention. The fourth patient had a pocket hematoma leading to prolongation of hospitalization by 1 day. No complications occurred after the 39 TV-ICD generator replacements.

### Mortality, Major Adverse Cardiac Events, and Cardiac Decompensation

**Figure 4.**
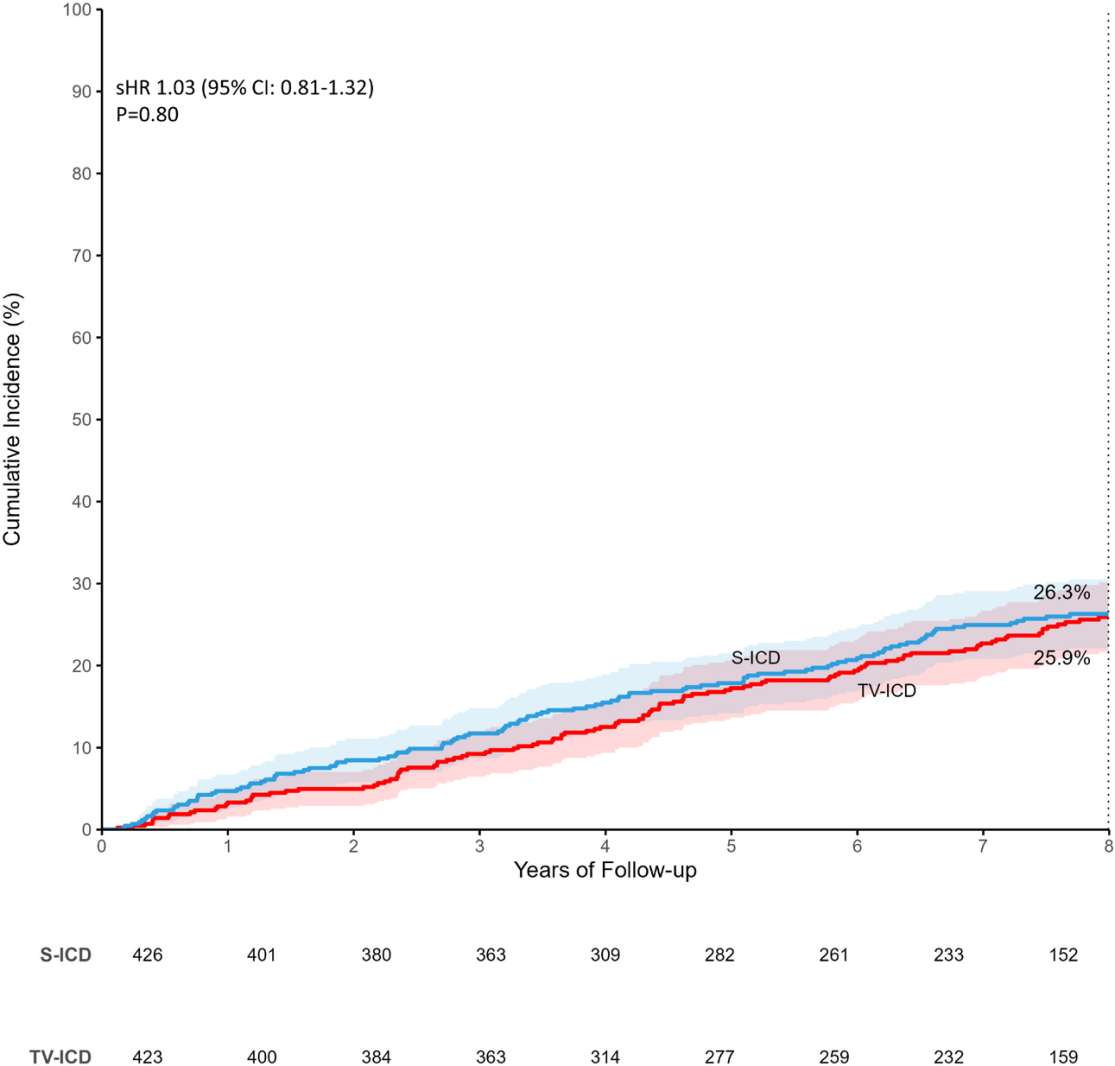
**Eight-year estimated cumulative incidence of all-cause mortality.** sHR indicates subdistribution hazard ratio; S-ICD, subcutaneous implantable cardioverter defibrillator; and TV-ICD, transvenous implantable cardioverter defibrillator.

The mortality rates were similar between study arms, with 125 deaths in the S-ICD group and 123 deaths in the TV-ICD group (8-year estimated cumulative incidence 26.3% and 25.9%, respectively; sHR 1.03 [95% CI, 0.81–1.32]; *P*=0.80; Figure 4). In the S-ICD group, the most common cause of death was noncardiovascular (54 of 125). In the TV-ICD group, the most common cause of death was other, nonsudden, cardiovascular death (49 of 123). Twenty-three patients in the S-ICD group died suddenly, and there were 27 sudden deaths in the TV-ICD group. Causes of death are presented in Table S6.

The number of patients with decompensated heart failure was similar between groups, with 105 patients in the S-ICD group and 99 in the TV-ICD group (8-year estimated cumulative incidence 24.5% and 21.7%, respectively; sHR, 1.06 [95% CI, 0.81–1.40]; *P*=0.67). Additionally, 103 patients in the S-ICD group and 114 patients in the TV-ICD group had a major adverse cardiac event (8-year estimated cumulative incidence 22.3% and 25.4%, respectively; sHR, 0.89 [95% CI, 0.68–1.15]; *P*=0.37).

## DISCUSSION

This primary analysis of the PRAETORIAN-XL trial demonstrated that, after a median follow-up of 87.5 months, there was no significant difference between the S-ICD and TV-ICD for all device-related complications. However, complications in the TV-ICD group were more often severe, with significantly more major complications leading to an invasive intervention. Besides, TV-ICD patients more often had a lead-related complication. Finally, mortality rates were similar between groups.

The modified intention-to-treat analysis did not show a significant difference in all complications (major and minor) between the S-ICD and TV-ICD. An earlier analysis of all complications in the PRAETORIAN trial showed that acute and late complications were equal after 48 months of follow-up.^8^ However, very late complications associated with transvenous leads, such as infection and lead failure, are expected to arise later on, as reported in earlier research, showing a 15% to 25% complication rate at 6 to 10 years.^11,12^ The PRAETORIAN-XL trial was conducted to analyze whether this rise in very late complications would lead to a difference between the S-ICD and TV-ICD. However, in PRAETORIAN-XL, the incidence of 11.6% at 8 years in the TV-ICD group was lower than expected. Besides this, only 25% of complications occurred during the extended follow-up, whereas previous research shows that chronic complications increase between 6 and 10 years.^12^ There are 2 reasons that could explain the discrepancy between our data and previous studies. The first explanation for a low event rate is the relatively low number of lead failures in our study. In previous studies, the Sprint Fidelis and Riata leads were still on the market, whereas in PRAETORIAN, these leads were not used anymore. The second explanation for the low event rate in the TV-ICD group is that the majority of patients in this group did not undergo a generator replacement during the study. Replacements expose patients to a new risk of procedure-related complications, and additional procedures almost double the risk of a device infection.^12^ Also, replacing the generator allows for re-evaluation of the initially implanted ICD, which can result in interventions to optimize device functionality. Last, in the S-ICD, a defibrillation test (DFT) is performed during most generator replacements. Failure of the DFT can lead to subsequent intervention, such as device or lead repositioning or change of device type. The generator replacement rate in the S-ICD arm of the trial was 5 times higher compared with the TV-ICD, partly because of the battery advisory field safety notice in S-ICDs. This contributes to the sudden rise in cumulative incidence of complications in the S-ICD at 5 years, which was not seen in the TV-ICD. Currently, the median service life of the S-ICD is 8.7 years, which might lead to fewer complications in the S-ICD with current and new devices because of less frequent generator replacements.^14^ On the other hand, the battery life of TV-ICDs is still longer, with a median of 10.8 years.^15^ Replacement procedures require a short hospitalization, which can be a burden to patients. The shorter battery life of the S-ICD, even though it does not lead to more complications, should therefore be discussed with every patient who receives this device.

### Major and Lead-Related Complications

Major complications occurred more frequently in the TV-ICD arm compared with the S-ICD arm. Additionally, lead-related complications were markedly more prevalent among patients in the TV-ICD arm, which is in line with findings from the ATLAS trial, which reported a >90% reduction in lead-related complications in the S-ICD compared with the TV-ICD.^16^ It underscores the severity of complications associated with transvenous devices, as most major complications in the TV-ICD arm were related to the lead. However, a transvenous lead gives the TV-ICD the ability to deliver brady- and tachycardia pacing, therapies the S-ICD cannot provide. This shortcoming could potentially be managed by the implantation of the recently introduced EMPOWER leadless pacemaker that communicates with the S-ICD and adds the option of ATP and bradycardia pacing to S-ICD therapy without the risk associated with transvenous leads.^17,18^ Although the benefit of ATP could be debated, as this does not lower the total amount of ICD shocks,^19,20^ and even though the EMPOWER pacemaker is not yet commercially available and long-term data on performance of this system are pending, this therapy might become a feasible alternative for patients who develop an indication for brady- or tachycardia pacing over time. In this way, patients who develop a pacing indication are not exposed to the risk of lead- and vein-related complications during the years of S-ICD therapy.

### Complications per Device Type

Many crossovers and upgrades to CRT-D occurred during the course of the study. These changes in device type were attributable to complications, progression of heart disease, sedation difficulties, or patient preference. As a result, 10 of 43 complications in the S-ICD arm actually occurred with a TV-ICD or CRT-D, and 2 of 55 complications in the TV-ICD arm occurred with a CRT-D. Of all device-related complications in this study, 58 of 98 (59%) were with a TV-ICD, whereas 33 of 98 (34%) were with an S-ICD. The as-treated analysis, which takes into account what type of device is actually implanted when a complication occurs, showed a significant benefit of the S-ICD compared with the TV-ICD with respect to all complications. These results should be considered in the evaluation of ICD therapy.

The number of patients who switched from an S-ICD to a TV-ICD during the study was more than twice as high as the number of patients who did the reverse, which might have been partly because of the emerging need for bradycardia pacing or the novelty of the S-ICD in the beginning of the PRAETORIAN trial. A proportion of the crossovers from the S-ICD to the TV-ICD may likely not occur in current clinical practice, such as crossover resulting from DFT failure or inappropriate sensing, which can be prevented or solved with correct device positioning.^21^ Nevertheless, as long as there is no commercially available leadless alternative for bradycardia pacing or CRT in S-ICD patients, a proportion of these patients will always require conversion to a transvenous device because of disease progression. Extracting an S-ICD and implanting a TV-ICD or CRT-D results in additional scarring and nonetheless exposes the patient to potential transvenous lead–related complications. However, venous access is preserved during the years with an S-ICD, and extraction of an S-ICD is associated with less risk than extraction of a transvenous device.^22,23^ As ICD therapy is often life long and might cover decades, these advantages and disadvantages should be considered for each individual patient.

### Future Perspectives

There are 2 considerations that remain important in the decision to implant an S-ICD or TV-ICD. The first consideration is the associated higher costs of the S-ICD compared with the TV-ICD. In the upcoming years, these costs might decline with arising competition from the extravascular ICD, which was introduced in 2023.^24^ The extravascular ICD consists of an extrathoracic generator on the left side of the thoracic wall and a lead in the substernal area, enabling pause prevention pacing and ATP, serving as an advantage over the S-ICD. Still, long-term clinical data on this device are limited. The second aspect is the need for sedation during the DFT at the S-ICD implantation procedure. The primary results of the PRAETORIAN-DFT trial, which randomized S-ICD patients to implantation with or without a DFT, will show whether the DFT can be omitted during future implantations.^25^

### Limitations

This trial has several limitations. First, the physicians who adjudicated the adverse events were not blinded for device type, but the randomization group was concealed. Second, a substantial number of patients did not consent to PRAETORIAN-XL and were therefore censored for further evaluation. However, patient loss was well balanced between study arms, and all modified intention-to-treat statistical models were corrected for loss to follow-up and death. Third, the power and sample size calculation of the initial PRAETORIAN trial was based on the composite end point of device-related complications and inappropriate shocks. The results of this extended PRAETORIAN-XL trial, which focused on complications alone, should therefore be interpreted with consideration of the original study design, which was not specifically powered for this isolated outcome. Fourth, as the risk of complications increases after a device intervention, this risk is not constant in time. The effect sizes reported in this article should therefore be interpreted as overall risk after 8 years and not as a constant hazard. Finally, the PRAETORIAN trial started in 2011, when overall experience with the S-ICD was limited compared with the TV-ICD. There is a significant learning curve associated with S-ICD implantation, and limited experience with implantation technique and follow-up with the S-ICD could have affected the complication rate in the S-ICD arm.^26^ In current clinical practice, the complication rate short after S-ICD implantation might be lower than shown in this trial because of increased experience with the device, limiting the number of complications as well as requiring less invasive interventions to solve complications. The 6-month complication rate of 2.4% in the ATLAS trial confirms this improvement.^16^

### Conclusions

The PRAETORIAN-XL trial demonstrated that, during long term follow-up, there was no significant difference between the S-ICD and TV-ICD in all device-related complications. However, the TV-ICD carries a significantly higher risk of major and lead-related complications compared with S-ICD therapy. The S-ICD should therefore be considered in all patients without a pacing indication who are evaluated for ICD therapy.

## Article Information

### Acknowledgments

The authors thank the patients who participated in this trial and the physicians and research coordinators for contributions to the conduct of this trial. Furthermore, the authors would like to express their gratitude to Jos Perdeck for his invaluable contribution to the statistical analysis of this study. J.A.d.V. has full access to all the data in the study and takes responsibility for its integrity and the data analysis.

### Sources of Funding

The PRAETORIAN-XL trial was funded by Boston Scientific, which had no role in the design of the trial, analysis of the data, or the drafting and submission of the manuscript (grants ISROTH20076 and ISRCAR00244).

### Disclosures

R.E.K. reports consultancy fees and research grants from Abbott, Boston Scientific, Medtronic, and Cairdac and has stock options from AtaCor Medical Inc. M.F.E. reports consultancy fees from Boston Scientific and Medtronic. S.M. reports consultancy fees from Boston Scientific and Medtronic. K.M.K. reports consultancy fees from Boston Scientific. P.D.L. reports educational and research grants from and is on the research board of Boston Scientific and reports research grants from Abbott. K.V. reports consultancy fees from Medtronic and Abbott. M.C.B. is a consultant for and receives honoraria as well as research grants from Boston Scientific and has equity in and is chief medical officer for AtaCor Medical, Inc. D.J.W. has consultancy arrangements with Boston Scientific, Medtronic, and iRhythm and a research grant from Boston Scientific. P.N. reports modest speaker honoraria from Biotronik, Boston Scientific, and Medtronic. M.A.M. reports consultancy fees from Boston Scientific. Z.I.W. is an advisor for Boston Scientific and is on the advisory board for Medtronic and Abbot, and reports speaker fees from Medtronic.

### Supplemental Material

Tables S1–S6

Figures S1 and S2

Additional statistical considerations and analyses

## Appendix

F.A.L.E. Bracke, MD, PhD, Department of Electrophysiology, Catharina Hospital, Eindhoven, the Netherlands;, J.G.P. Tijssen, PhD, Amsterdam UMC, University of Amsterdam, Heart Center, Department of Cardiology, Amsterdam Cardiovascular Sciences Heart Failure & Arrhythmias, Amsterdam, The Netherlands;, T. Demming, MD, Department of Internal Medicine and Cardiology, University Medical Center Schleswig-Holstein, Kiel, Germany;, F.J. Oosterwerff, MD, PhD, Department of Cardiology, OLVG, Amsterdam, the Netherlands;, F. Leyva, MD, PhD, Department of Cardiology, Queen Elisabeth Hospital Liverpool, Liverpool, United Kingdom;, T. Germans, MD, PhD, Department of Cardiology, Medisch Centrum Alkmaar, Alkmaar, The Netherlands;, J. Perdeck, MSc, Amsterdam UMC, University of Amsterdam, Heart Center, Department of Cardiology, Amsterdam Cardiovascular Sciences Heart Failure & Arrhythmias, Amsterdam, The Netherlands.
